# Smart e-Textile Singlet Prototype and Concept: Multi Sensor Sensing for Geriatric Monitoring

**DOI:** 10.3390/bioengineering12111275

**Published:** 2025-11-20

**Authors:** Tobias Steinmetzer, Florian Wieczorek, Anselm Naake, Peter Wolf, Alexander Braun, Sven Michel

**Affiliations:** 1 Department of Therapy Sciences (II), Brandenburg University of Technology (BTU) Cottbus-Senftenberg, Platz der Deutschen Einheit 1, 03013 Cottbus, Germany; alexander.braun@b-tu.de (A.B.); sven.michel@b-tu.de (S.M.); 2 Smart Textiles Hub GmbH, Königsbrücker Straße 96, Halle 16C, 01099 Dresden, Germany; 3 Fibercheck GmbH, Technologie-Campus 1, 09126 Chemnitz, Germany

**Keywords:** singlet, Smart e-Textile, wearable, IMU sensors, ECG, blood pressure, breathing

## Abstract

This paper explores the development of a Smart e-Textile Singlet designed to enhance geriatric care through continuous monitoring of vital health parameters. The proposed garment integrates various sensors to measure core body temperature, blood oxygen saturation, respiration rate, blood pressure, pulse, electrocardiogram (ECG), activity level, and risk of falls. Leveraging advanced technologies such as inertial measurement unit (IMU) sensors, thermoelectric materials, and piezoelectric fibers, the e-textile ensures both functionality and sustainability. Additionally, artificial intelligence algorithms are employed to provide near-real-time feedback and early warnings, significantly improving health management for elderly individuals. This innovative approach not only promotes autonomy and well-being among the elderly but also alleviates the workload of healthcare providers. The Smart e-Textile Singlet represents a multi-sensor solution by offering a holistic monitoring system.

## 1. Introduction

The field of geriatrics has significantly evolved over the past few decades to meet the needs of an aging population. Modern approaches in geriatric medicine include innovative technologies, advanced care concepts, and comprehensive strategies to promote the health and quality of life of elderly individuals. The use of telemedicine and digital health solutions has increased significantly in geriatrics. These technologies enable elderly patients to access medical advice and care without leaving their homes. Telemedicine platforms offer regular monitoring of chronic diseases, virtual consultations, and even psychological support [1]. Assistive technologies, such as wearable health monitors, intelligent emergency systems, and robotic assistance, play a crucial role in enhancing the safety and independence of elderly people [2]. Modern care models integrate health and social services to provide comprehensive support. Programs like “Aging in Place” allow elderly individuals to remain in their familiar environment while having access to medical care, home care, and social services. These integrated approaches promote the autonomy and quality of life of elderly people [3]. Preventive measures and health promotion are central to modern geriatrics. Programs that promote physical activity, healthy eating, and social interaction contribute significantly to maintaining the health and well-being of elderly individuals. Senior sports groups, nutrition courses, and social clubs are examples of such initiatives [4].

Smart e-Textiles are often equipped with multiple sensors to capture various physiological and movement parameters. However, their capabilities are often limited by factors such as the restricted number of measurement channels, insufficient signal quality due to textile–skin contact issues, high energy consumption, or limited data processing and storage capacity. In most cases, a wireless connection to a smartphone is used to visualize or transmit the collected data. The MagIC system is a vest. A 1-channel ECG, a textile-based piezoresistive plethysmograph for measuring respiratory rate and an acceleration sensor are integrated in the vest [5].

Nettextiles has developed an arm sleeve equipped with IMU sensors to measure arm movement in order to optimize movement [6]. Hexoskin is a vest which is equipped with electrodes for ECG recording and stretch sensors for measuring respiration [6]. Several commercial smart textiles, such as Hexoskin or Nettextiles, are already available on the market. However, these systems are often limited in their functionality. They typically focus on a small number of physiological parameters, such as heart rate or respiration, and lack integrated motion analysis or context-aware sensing. Moreover, most systems require external modules for data storage and processing, which restricts their usability in everyday life. In contrast, the smart textile presented in this paper integrates multiple physiological and kinematic sensors in a single, comfortable garment and processes the data directly on the embedded unit, enabling comprehensive and continuous health monitoring.

Zhang et al. provide a comprehensive review of wearable artificial-intelligence driven biosensor networks, highlighting how high-dimensional data processing enables non-invasive monitoring of physiological parameters and anticipatory diagnostics [7]. In our system, we similarly envisage the use of AI techniques for multimodal sensor fusion and adaptive health monitoring.

For example, the review by Chen et al. discusses how nanomaterials in 3D-printed wearables can significantly enhance sensor integration and flexibility, which is highly relevant for textile-based health monitoring systems [8]. In our work, by contrast, we adopt a smart textile approach rather than 3D-printed wearables, yet the emphasis on material innovation and seamless integration remains similarly critical. It may prove beneficial at a later stage to combine these two approaches. Recent developments in microscale sensing highlight the role of MEMS technologies as a “sensory nervous system” for embodied AI agents, enabling highly integrated, low-power multimodal perception systems [9]. In the present work, the smart textile extends this paradigm by embedding multiple physiological and kinematic sensors into a single wearable platform—paralleling the move from discrete modules to integrated sensory systems discussed by Zhou et al. [9].

## 2. Materials and Methods

**Figure 1:**
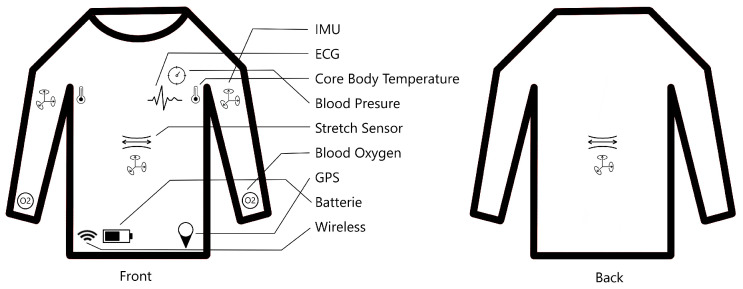
Shematic of the Smart e-Textile Singlet with functions for pulse, blood pressure, risk of falling, position, ECG, activity level, respiratory rate, oxygen saturation in the blood, core body temperature, and GPS.

For this reason, we are researching a Smart e-Textile Singlet. The concept of the textile is shown in Figure 1. Sensors will measure core body temperature, blood pressure, blood oxygen saturation, respiration rate, pulse, ECG, activity level, and risk of falls. These measurements are intended for prophylactic use.

The activity level is used preventively to help the subject adjust their daily routine according to their physical capabilities. To simplify everyday life, vital parameters are recorded continuously. This approach can relieve nursing staff by reducing the manual workload associated with data collection. A key aspect of the system lies in fall prevention through holistic physiological monitoring. Instead of detecting single events, the Smart e-Textile aims to capture the overall physical condition of the wearer. Parameters such as body temperature, heart rate, blood oxygen saturation, respiratory pattern, step cadence, and gait symmetry together form an individualized health profile. Changes in these indicators often precede critical events such as weakness, dizziness, or balance loss. By continuously monitoring and correlating these vital signs and motion features, the system can identify gradual deterioration in the user’s condition—for example, signs of fatigue, circulatory instability, or reduced coordination. When deviations from the individual baseline are detected, the textile can issue early warnings, allowing preventive action before a fall occurs. This approach transforms fall prevention from a reactive process into a proactive health management strategy, using objective sensor data to detect early warning signs of instability. The Smart e-Textile thereby supports both the wearer and healthcare professionals by providing continuous, data-driven insights into the person’s well-being. As a clinical benchmark, the Tinetti Test—a widely used assessment tool for balance and gait in older adults—will serve as a reference for validating the accuracy and clinical relevance of the system’s fall risk detection [10].

**Figure 2:**
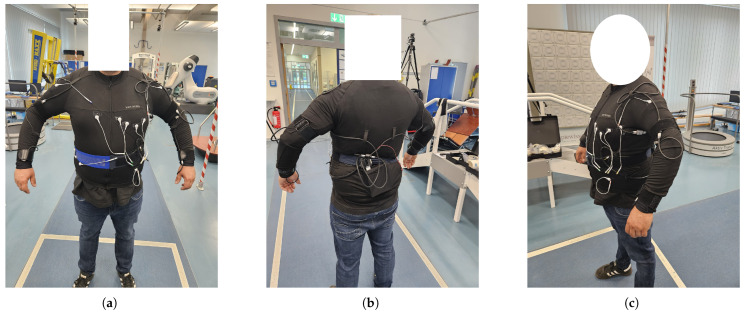
Prototype of the Smart e-Textile Singlet with functions for pulse, blood pressure, risk of falling, position, ECG, activity level, respiratory rate, oxygen saturation in the blood, core body temperature, and GPS. In (**a**) you can see the front of the singlet. (**b**) The back of the singlet. (**c**) A side view.

The power supply for the smart textile may be implemented using thermoelectric materials or piezoelectric fibers integrated into the fabric [11]. The current prototype, as shown in Figure 2, was developed and manufactured by Smart Textiles Hub GmbH.

The vital signs collected are processed using a combination of machine learning and deterministic algorithms to assess the user’s physiological condition. For instance, convolutional recurrent neural networks (CRNNs) are planned for activity recognition and respiration classification, while convolutional neural networks (CNNs) have proven effective for ECG analysis [12]. Deterministic algorithms with defined thresholds are applied for temperature monitoring. In addition, sensor fusion methods will be implemented to combine data from stretch and IMU sensors, enabling a more comprehensive interpretation of body movement and respiration dynamics. If parameters such as pulse, or ECG rise sharply, this can be a possible indication that the person is feeling significantly worse and needs to take a break during activity, for example. The sensors used to create the prototype are shown in Table 1.

The vital signs collected are used to show the artificial intelligence the person’s current condition. If parameters such as pulse, blood pressure or ECG rise sharply, this can be a possible indication that the person is feeling significantly worse and needs to take a break during activity, for example.

### 2.1. Posture-Adaptive Core Temperature Estimation

Accurate monitoring of core body temperature is crucial in geriatric health surveillance. In this Smart e-Textile Singlet, a temperature sensor Measurement Specialties, Inc., Paris, France, HTU21 is placed in the axillary region (under the left armpits), a common and clinically accepted location for non-invasive core temperature estimation. However, the accuracy of axillary temperature readings heavily depends on arm position. If the arm is lifted or held away from the torso, the sensor becomes exposed to ambient air, leading to an underestimation of the true core temperature [13,14].

To address this, we integrate inertial measurement units (IMUs) on the upper arms and the thoracic spine. These allow us to determine the orientation of each arm relative to the spine and ensure that the arm is sufficiently adducted (close to the body) during temperature measurement, which recorded at a frequency of 10 Hz. We use the Bosch BNO085 IMU sensor, which samples at 250 Hz and provides both linear acceleration and quaternion data. The BNO085 includes an embedded sensor fusion algorithm based on an internal Kalman filter, which computes absolute orientation (quaternion) directly on the chip. By comparing the orientation of the upper arm IMUs with that of the spine IMU, we derive the arm–spine angle.

This biomechanical filtering is supported by literature showing significant degradation in axillary temperature accuracy when the arm is not adducted. Nathansen et al. [14] demonstrated that lifting the arm for one minute can reduce axillary readings by up to 1.9 °C compared to core values. Taylor [13] found close agreement with esophageal temperature only when the arm was fully adducted. Niven et al. [15] and Hashimoto et al. [16] reported high correlations between axillary and core measurements when proper arm positioning was ensured [17].

In conclusion, the IMU-based posture classification enhances the reliability of core body temperature measurements. By ensuring that only readings taken under physiologically valid conditions are accepted, we improve both the safety and diagnostic value of wearable thermometry in elderly patients.

### 2.2. ECG Sensors

The Smart e-Textile Singlet is equipped with an eight-channel ECG system based on the ADS1298 analog front-end by Texas Instruments, Dallas, TX, USA, which is specifically designed for biopotential measurements in wearable medical devices. The ADS1298 offers multiple differential input channels, low noise, and low power consumption, making it suitable for continuous, long-term monitoring in ambulatory settings [18].

For electrode placement, we employ a reduced-lead configuration adapted from the standard twelve-lead ECG system, optimized for integration into a non-personalized textile layout. Electrode positions are selected to balance clinical relevance with the practical constraints of standardized shirt sizing. ECG signals are recorded at a sampling frequency of 250 Hz.

- Two electrodes are placed on the left and right forearms (limb leads).
- Two electrodes are placed on the left and right lower torso, roughly at the anterior superior iliac spine.
- V1: Fourth intercostal space, right of the sternum.
- V2: Fourth intercostal space, left of the sternum.
- V3: Midway between V2 and V4.
- V4: Fifth intercostal space at the midclavicular line.
- V5: Same horizontal level as V4, at the anterior axillary line.
- V6: Same horizontal level as V4, at the midaxillary line.

Due to the use of standard shirt sizes and non-individualized electrode placement, deviations from ideal clinical positions are expected. During the validation phase, we will assess the signal quality of each channel. Channels that consistently deliver low signal quality or are redundant may be omitted in the final design, reducing system complexity and power consumption while preserving diagnostic utility.

### 2.3. Blood Pressure

Blood pressure measurement is extremely important for the assessment of a person’s cardiovascular health. Using a blood pressure monitor with an upper arm cuff is still the most common method. However, this device has serious limitations—it only provides a static pair of blood pressure readings, it cannot detect fluctuations in blood pressure over time, it is inaccurate, and it is uncomfortable to use [19].

Non-contact blood pressure measurement using CW Doppler radar is a new method that will most likely replace old techniques. The principle is that tiny displacements on the body surface caused by the central aortic artery are detected by a digital IF-CW Doppler radar. This allows the pulse transit time (PTT) to be extracted in order to estimate blood pressure [20]. For this reason, we plan to integrate the Infineon radar chip for blood pressure recording in future development stages [21].

In the current prototype, no sensor for blood pressure measurement is integrated yet; however, its inclusion is planned for future versions of the smart textile system.

### 2.4. Blood Oxygen Saturation

The oxygen level in the blood is a key indicator of the well-being of patients with heart or lung conditions, those undergoing anesthesia, or infants under observation [22]. For this purpose, we integrate a photoplethysmography (PPG) sensor into the textile system to enable continuous, non-invasive monitoring of peripheral blood oxygen saturation (SpO_2_).

We employ the MAX30102 sensor, a compact optical biosensor that combines two LEDs (red and infrared) with a photodetector to measure SpO_2_ and heart rate. The sensor is placed on the underside of the forearm, a location chosen to balance signal quality with wearability and textile integration. Data are recorded at a frequency of 4 Hz, which is sufficient for reliable tracking of physiological changes under resting and low-motion conditions.

Several commercial systems have demonstrated the feasibility of forearm-based SpO_2_ monitoring [23], although motion artifacts and ambient light interference remain challenges in wearable applications. The integration of the MAX30102 in our design leverages its low-power consumption, digital I^2^C interface, and onboard signal processing, making it well suited for long-term, textile-based health monitoring in geriatric settings.

### 2.5. IMU Sensor

Inertial measurement units (IMUs) are strategically placed on the upper arms, underarms, chest, and spine to capture a range of motion and physiological parameters. We use the Bosch BNO085 sensor, which samples at a frequency of 250 Hz and provides linear acceleration and quaternion data. The sensor features an internal Kalman filter for real-time sensor fusion, delivering accurate orientation estimates directly on the chip.

The IMU sensors on the upper arms are primarily used to record arm movements. From this, motor symptoms, general activity levels, and potential fatigue indicators can be derived [24]. Additionally, these sensors contribute to verifying correct arm posture during body temperature measurement to ensure the validity of axillary readings (see Section 2.1). The chest-mounted IMU captures thoracic expansion and allows for continuous respiratory rate estimation [25]. These signals will be validated using a reference motion capture system (Vicon) to ensure accuracy [26]. Another IMU is attached to the spinal column, aligned with the chest IMU. This sensor serves two main purposes. First, it helps isolate thoracic movement from abdominal interference, particularly during ambulation, thereby enabling robust respiration monitoring while walking. Second, it is used for gait analysis, providing clinically relevant parameters such as balance, walking speed, and trunk inclination. Additionally, the spinal IMU is intended to run an embedded activity classification model, capable of distinguishing between basic postures and activities: sitting, lying, walking, running, and stair climbing. This enables context-aware interpretation of other physiological signals (e.g., temperature, SpO_2_), enhancing diagnostic relevance.

### 2.6. Stretch Sensor for Respiratory Monitoring

In addition to inertial sensing, we integrate a stretch sensor into the textile to capture thoracic expansion during respiration. The sensor is positioned across the chest area to detect circumferential changes associated with inhalation and exhalation. Stretch-based measurements offer a direct mechanical signal of chest wall movement and have proven effective in wearable respiratory monitoring systems.

The stretch sensor is a smart textile, which essentially behaves like a variable resistor depending on its elongation. Structurally, it is a weft-knitted band composed of interlaced loops (stitches) rather than straight yarns as in woven fabrics. A non-conductive yarn forms the basic layer, arranged in knit rows and columns. During fabrication, a conductive yarn is “plated” alongside the non-conductive yarn in selected rows to form a meander-shaped structure.

Because the conductive yarn is not insulated, electrical short circuits occur at the loop intersections in the unstretched state. As the textile stretches, the loops straighten, reducing the number of contact points between conductive segments. This results in an increased electrical resistance, since the current must travel a longer, uninterrupted path through the sensor. This unique structural behavior allows the fabric to transduce mechanical strain into an electrical signal sensitive to chest expansion.

To improve the accuracy and robustness of respiratory rate estimation, we aim to develop a sensor fusion algorithm that combines data from the stretch sensor with the IMU on the chest. While the stretch sensor provides high sensitivity to expansion, it can be affected by motion artifacts during ambulation. Conversely, the chest IMU offers detailed motion dynamics that can be used to distinguish between respiratory motion and other body movements.

By combining these complementary signals—mechanical strain from the stretch sensor and orientation/acceleration from the IMU—we aim to enhance respiration detection under both resting and dynamic conditions. This multimodal approach is expected to improve signal reliability, especially in real-life scenarios where users are not stationary.

Physiologically, a normal respiratory rate ranges from approximately 12 to 40 breaths per minute, depending on age, activity level, and health status. This corresponds to a frequency range of from about 0.2 Hz to 0.67 Hz, which must be taken into account during signal processing and analysis. Consequently, signal filtering and frequency-domain methods—such as bandpass filtering or spectral analysis—should be tuned to this range to reliably extract respiratory patterns while suppressing noise and non-respiratory motion [27].

To satisfy the Nyquist-Shannon sampling theorem, the sampling frequency of the sensor system must be at least twice the highest expected respiratory frequency component. Given a physiological upper bound of 0.67 Hz (40 breaths per minute), the minimum required sampling rate is approximately 1.34 Hz. However, in practice, significantly higher rates (e.g., 10–50 Hz) are used to ensure accurate reconstruction of the respiratory signal, to detect subtle dynamics such as changes in breathing depth, and to facilitate artifact detection and filtering. This oversampling provides a more robust foundation for feature extraction and fusion-based analysis.

### 2.7. Power Supply

The smart singlet employs a hybrid power system combining flexible thermoelectric generators, and piezoelectric fibers. Photovoltaic cells integrated into the shoulder region harvest ambient light, while thermoelectric modules convert body heat into electrical energy. Piezoelectric fibers woven into the textile generate energy from biomechanical motion (e.g., walking or arm movements). A 50 mAh LiPo battery serves as a backup, ensuring continuous operation during low-energy conditions. This approach aligns with recent advances in textile-based energy harvesting [11,28].

## 3. Ethical Approval

The study protocol for the development and validation of the smart undershirt was submitted to the Ethics Committee of Brandenburg University of Technology Cottbus–Senftenberg and received a positive vote on 30 August 2024 (reference number: EK2024-26). The investigation involves healthy adult participants and follows a four-part experimental design.

The present dataset consists of recordings from a total of 21 participants, of which 7 were men and 14 were women, with an average age of 22.29 years (σ=2.83 years).

Initially, vital parameters such as electrocardiogram (ECG), blood pressure, heart rate, core body temperature, respiratory rate, thoracic expansion, and oxygen saturation are recorded while the participants remain at rest. This is followed by a gait and activity analysis, during which the participants walk a 10-meter path back and forth. A Vicon motion capture system and the Locometrix system are employed for data validation.

Subsequently, participants complete a standardized movement sequence including sitting, lying down, walking, running, and stair climbing to evaluate sensor performance under typical daily activities. In the final phase, participants perform exercises such as rowing, bench pressing, push-ups, and bicep curls until the onset of muscle tremors. This phase captures physiological responses during increasing fatigue, supported by additional inertial sensors on the upper limbs. Rowing is used as a specific warm-up to minimize the risk of injury.

Upon completion, participants filled out a structured questionnaire to evaluate the comfort, usability, and overall perception of the smart undershirt. The questionnaire consisted of twelve items addressing aspects such as perceived autonomy in data handling, feeling of being observed, behavioral changes during wearing, number of sensors and perceived discomfort, sense of compression, potential skin irritation, material comfort, ease of putting on the garment, freedom of movement, constant perception of the sensors, thermal comfort (feeling cold or sweating), and breathability of the fabric. Each item was rated on a 10-point Likert scale, with 10 indicating the most positive experience and 0 the least favorable. The feedback obtained from the questionnaire will inform future improvements in design, material selection, and sensor integration, ensuring enhanced user comfort and acceptance of the smart textile system.

## 4. Results

### 4.1. Questionnaire

The results of the user questionnaire (Table 2) show an overall positive user experience with the smart undershirt. Items marked with an asterisk (*) were inversely scaled, meaning that lower values correspond to better ratings. Participants reported that the material was pleasant (7.94±1.51) and that usual movements could be performed without restrictions (7.78±2.05). The undershirt was also easy to put on (6.33±2.00) and provided satisfactory thermal comfort (6.22±2.58). Inversely scaled items such as perceived compression (2.17±0.51), feeling of being observed (3.50±2.31), and discomfort (3.44±2.20) indicate that most participants did not experience these effects strongly. Overall, the feedback suggests good acceptance of the prototype, with minor potential for improvement in terms of perceived sensor presence and breathability.

Nevertheless, personal feedback collected during informal interviews with participants indicated that the current prototype still requires improvement. Some participants reported that the external cable routing occasionally interfered with natural arm movement, especially when using handrails during stair climbing. This observation suggests that while the participants recognized the potential of the system, the current prototype is not yet fully optimized for unrestricted daily use. At this stage, the cable harnesses were intentionally routed externally to allow easy maintenance and modification during testing. In future iterations, these components will be fully integrated into the textile structure to enhance comfort and usability.

### 4.2. Temperature Signal

To identify periods of thermal stability, the temperature signal was analyzed using a custom algorithm that detects time intervals with minimal variation over a defined duration. This approach allows for an objective assessment of stable temperature phases within the measurement sequence.

**Figure 3:**
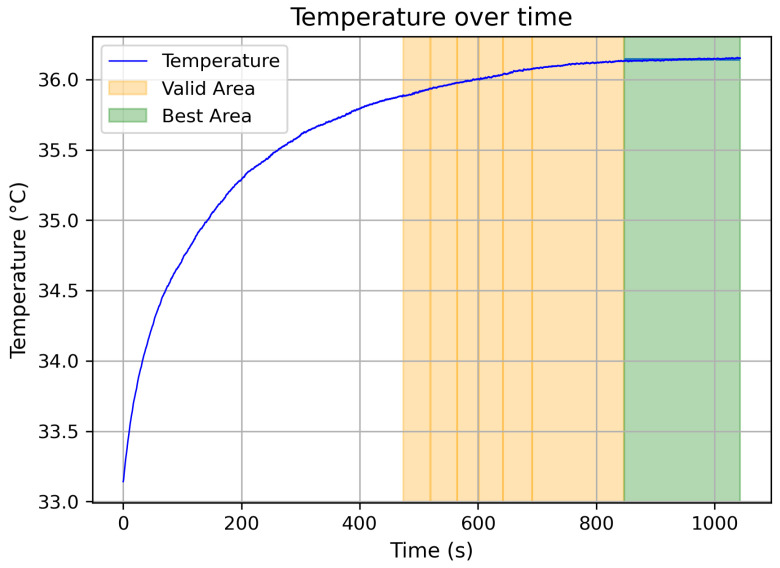
Smoothed temperature signal with automatically detected stable regions (green: best stable interval).

Figure 3 illustrates an example of a smoothed temperature curve with highlighted stable regions.The interval with less varianz was used for further evaluation, providing a mean temperature of 36.142 °C with a standard deviation of 0.009 °C. These values confirm the reliability of the temperature sensing unit under controlled test conditions.

Furthermore, it can be observed that the stable region occurs relatively late, starting at approximately 850 s. This behavior is attributed to the gradual heating of the temperature sensor from ambient to body temperature. During continuous daily wear, it is expected that the sensor will reach and maintain a stable temperature more quickly, resulting in improved measurement consistency.

### 4.3. Inertial Measurement Unit (IMU) Data

**Figure 4:**
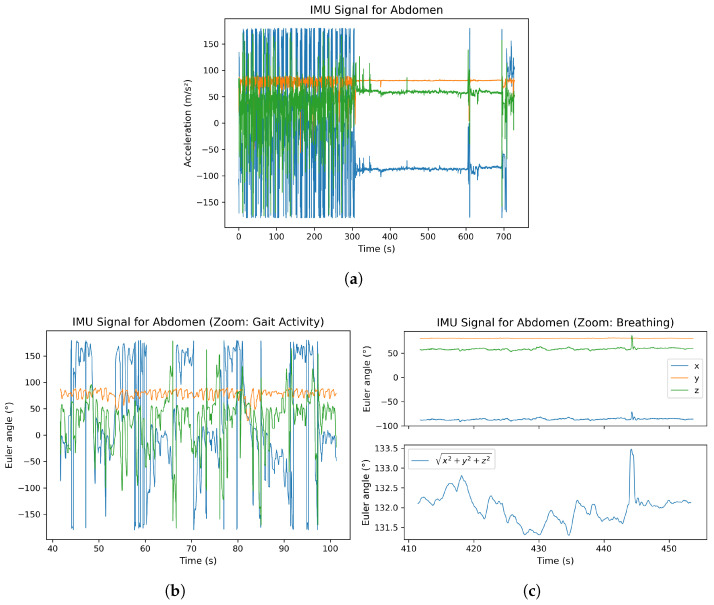
Raw Euler angle signals from the chest-mounted IMU sensor: (**a**) complete measurement with gaot activity until 300 s, after that a resting phase; (**b**) magnified gait sequence showing step-related oscillations; (**c**) resting phase with visible respiration-induced motion and computed signal magnitude.

In this section, the raw Euler angle data of an IMU sensor are presented as an example. The sensor placed on the chest was selected as a representative case. Figure 4 is divided into three subplots for better clarity. In subplot (a), the complete signal of the IMU sensor located at the abdomen is shown. Up to approximately second 300, the participant performed a walking activity, which explains the significantly higher amplitudes observed in the signal. Subplot (b) provides a magnified view of the walking sequence. Particularly in the *y*-axis, clear sinusoidal oscillations can be identified, corresponding to the participant’s individual steps. Subplot (c) illustrates the resting phase of the experiment. In this section, the respiratory signal is clearly visible. In the lower part of subplot (c), the signal magnitude was additionally computed as follows:

(1) $$e=\sqrt{x^{2}+y^{2}+z^{2}}$$

to highlight the overall dynamic behavior of the sensor during respiration. The resulting magnitude signal reveals periodic variations that coincide with the participant’s breathing cycles, demonstrating the IMU’s sensitivity to subtle thoracic movements.

## 5. Discussion

### 5.1. Technical Feasibility

The integration of multiple textile-based sensors into a comfortable undershirt is technically achievable and has been demonstrated in recent prototypes. For example, a prototype cardio-respiratory monitor embedded four conductive textile strain sensors for breathing and a single IMU for heart-rate monitoring into a lightweight wearable, achieving reliable vital-sign estimates across sitting, standing, and supine postures [29]. Similarly, smart garments have been developed by embedding conductive fiber fabrics, leads, and electronics into clothing to enable Holter-style ECG monitoring [30]. Such designs use embedding or coating methods (rather than bulky modules) to preserve comfort and washability: embedding sensors within fabric threads or coatings yields durable, stretchable electronics with minimal impact on garment feel [31].

Nevertheless, integration poses challenges: secure contact and alignment of textile electrodes or stretch sensors is crucial. In textile ECG tests, dry fabric electrodes recorded P, QRS, and T waves comparable to conventional gel electrodes at rest, but motion artifacts increased during vigorous movement [30]. In one study, a single-lead textile ECG shirt remained usable for daily monitoring but failed under vigorous trunk twisting [30]. This highlights the need for robust attachment (e.g., snug fit or dynamic straps) and signal conditioning to reduce noise.

Power management is another key concern: miniature batteries can be complemented by textile energy harvesters. Recent work on triboelectric nanogenerators (TENGs) shows that flexible, fabric-based TENGs can convert body movements (bending, stretching, tapping) into electrical energy [28]. Textile TENGs knitted into clothing have been used to harvest biomechanical energy from respiration and motion, offering a self-powered option [28]. In practice, a hybrid approach of batteries plus intermittent energy harvesting may extend device uptime. Overall, the combination of conductive threads, printed/stretchable electronics and textile-based power modules appears feasible, but must balance signal quality, form factor, and wash durability (e.g., one textile ECG electrode retained function over dozens of machine washes [30]). Long-term use in geriatric settings will require user-friendly design (no rigid components) and energy-efficient operation, but early prototypes demonstrate that integrated, multi-sensor smart garments can deliver high-quality vital signals in everyday environments [29,31].

### 5.2. Clinical Relevance

Continuous vital-sign monitoring in older adults can greatly enhance care by enabling early detection of deterioration and supporting independent living. Wearable sensors allow health metrics to be tracked outside the clinic, which is vital for vulnerable elders with chronic conditions or mobility limitations [32,33]. For example, continuous ECG, SpO_2_, temperature, and respiratory data can detect arrhythmias or hypoxemia that periodic checks might miss. As one review notes, remote monitoring systems are especially advantageous for older adults and those with chronic diseases, as they reduce hospital burden while improving access to timely care [34,35].

Wearables can also enable clinicians and caregivers to intervene early: clinicians can remotely review up-to-date vital signs and even conduct video visits, supporting self-management of stable conditions in the home [33,34]. This can improve health outcomes by catching subtle trends (e.g., rising heart rate or falling SpO_2_) before an acute crisis.

Fall prevention is another critical application. Falls are the leading cause of accidental injury and death in those over 65 [36], and many falls result from underlying issues like gait instability or sudden cardiovascular events. Wearable IMUs and pressure sensors can continuously assess balance and gait patterns. Fall-risk algorithms using IMU data have been shown to distinguish high-risk gait features and classify fallers with high accuracy (e.g., ∼89% using support-vector machines on gait metrics [37]). Coupling vital signs with motion data could offer even earlier warnings (for instance, detecting sudden heart-rate changes that precede a syncopal fall). In sum, continuous monitoring provides a richer picture of an elder’s daily status: alerting caregivers to emergencies (falls, arrhythmias) and enabling preventive interventions (medication adjustment, balance training) well before hospitalization is needed [36,38,39].

For caregivers and physicians, this means better situational awareness and data-driven decision-making. Rather than relying solely on infrequent checkups or manual logs, clinicians gain access to objective trends over time, and families can be notified of significant events. In practice, pilot programs have shown that elderly users are willing to wear sensors and that families value the security and data these systems can provide [33,35]. By linking sensor data to telehealth and home care platforms, smart garments can augment routine care and empower elderly patients, improving quality of life while alleviating strain on healthcare resources [32,35].

### 5.3. Limitations

Despite these promises, several limitations must be acknowledged. Sensor accuracy can suffer from misplacement and motion artifacts: for example, even high-quality textile ECG electrodes pick up noise when the wearer twists or shifts, leading to false readings if uncorrected [30]. Similarly, stretch sensors can lose calibration if the garment rides up or compresses unpredictably. Careful sensor placement and frequent recalibration may be needed in long-term use. In addition, signal noise from muscle activity or environmental interference can confound measurements (e.g., ECG baseline wander during movement). Algorithms must filter artifacts without discarding clinically important variations.

Validation in real-world elderly populations is another challenge. Most prototype tests involve small numbers of healthy volunteers under controlled conditions [29,30]. Older adults have diverse body shapes, skin conditions, and comorbidities (e.g., tremors, arthritis) that could affect sensor contact and algorithm performance. Clothing fit, skin moisture, and movement patterns vary widely among seniors, so rigorous testing is needed across representative subgroups. Frail or cognitively impaired patients might also wear the garment incorrectly or intermittently. In terms of algorithm generalizability, age-related changes (e.g., lower heart rate variability) require retraining models on elderly datasets to avoid bias.

Finally, ethical and privacy issues are paramount. Continuous tracking of location, heart rhythms, and activity generates sensitive data. Robust encryption and secure data handling are essential to protect patient privacy. Wearable systems must comply with healthcare data regulations (HIPAA, GDPR) and ensure that only authorized caregivers see alerts. Moreover, an equity gap exists in access: not all older adults have high technology literacy or internet access [32]. Systems should be designed with user-centered interfaces and support for low-tech users. The digital divide may otherwise limit adoption in disadvantaged groups. In summary, while smart undershirts can produce clinically useful data, care must be taken to validate performance in the intended population and to address security, consent, and accessibility concerns [32,35].

### 5.4. Future Work

Future steps include extensive pilot studies and clinical trials with elderly users. Prototype garments should be tested in home and care settings to assess usability, adherence, and clinical impact. These trials can also refine sensor placement and garment design based on user feedback. Parallel work on AI models is crucial: machine learning can analyze the continuous data streams to detect anomalies or classify activities. For instance, existing fall-detection systems using wearable IMU data have achieved high accuracy by training SVM and neural-network classifiers on gait and motion features [37]. Similar techniques can be developed to flag deterioration in vital signs or unusual behaviors (e.g., prolonged inactivity). Anomaly-detection algorithms, possibly based on deep learning or hybrid rule-based models, could provide early warnings of heart failure decompensation, infection, or other events.

Finally, integration into healthcare workflows is needed. The smart undershirt system should interface with telehealth platforms and electronic medical records so that sensor data and AI-generated alerts are visible to clinicians and caregivers. User-friendly dashboards or apps can present trends and flags to healthcare providers. Embedding alerts into caregiver workflows (e.g., mobile alerts or nurse dashboards) will help translate data into action. In the broader context, connecting these garments to remote monitoring programs aligns with the push for “smart health” technologies [32,34]. By pursuing these development steps—iterative trials, AI model refinement, and telehealth integration—this smart undershirt concept can move towards real-world deployment as a tool for elder care and fall prevention.

## 6. Conclusions

The development and implementation of a smart e-textile for monitoring the health and activity levels of elderly individuals represent a significant advancement in geriatric care. By integrating various sensors into a wearable garment, we aim to provide continuous, non-invasive monitoring of vital parameters such as core body temperature, blood oxygen saturation, respiration rate, blood pressure, pulse, ECG, activity level, and risk of falls. This innovation does not only enhances the quality of care but also promotes the autonomy and well-being of elderly individuals by enabling proactive health management.

Our approach leverages cutting-edge technologies such as IMU sensors, ECG, stretch sensors which collectively ensure the functionality and sustainability of the smart textile. The inclusion of artificial intelligence further augments the system’s capability to provide real-time feedback and early warnings, thus preventing potential health issues before they escalate.

The integration of the presented multi sensor system and technologies can help to consolidate the numerous products currently on the market into a single product capable of storing all recorded vital parameters in a shirt [5,6]. This relieves the burden on caregivers, enabling better patient care in geriatrics. Furthermore, algorithms can help to process the vital parameters effectively. For example, in fall prevention, vital parameters can be used to gain a better overall picture of the patient and thus react more effectively to critical values. This leads to a better quality of life for patients.

In summary, the proposed smart e-textile is a promising innovation in geriatric care by combining multiple health monitoring functions into a single, wearable garment. The system’s ability to provide continuous monitoring and real-time feedback will play a crucial role in enhancing the safety, independence, and quality of life for elderly individuals. As we move forward, further research and validation will be essential to ensure the effectiveness and reliability of this technology in real-world settings.

## Figures and Tables

**Table 1:** Overview of the sensors, measured quantities, physical limits, and frequency.

Sensor	Measured Quantities	Physical Range/Limits	Frequency
ECG (ADS1298)	24-bit data for 8 channels in voltage	Sample rate up to 32 kSPS	250 Hz
Temperature (SHT21/HTU21)	Temperature, relative humidity	−40 °C to +125 °C, 0% to 100% RH (non-condensing)	6 Hz
Blood Oxygen (MAX30102)	Red and infrared light absorption, measured and converted via algorithm into pulse rate and oxygen saturation (SpO_2_). Sampling rate is 100 Hz. SPO_2_ values are averaged over 20 raw samples.	SpO_2_: 70–100% (below 70% unreliable/invalid), Heart rate: approximate 30–240 BPM	6 Hz
GPS (NEO-6MV2)	Longitude and latitude for position tracking	Horizontal accuracy 2.5–5 m, vertical accuracy usually 5–10 m	6 Hz
IMU (BNO08x)	i, j, k (quaternion vector components), real (scalar part), accuracy (uncertainty in radians), ax, ay, az (linear acceleration without gravity, in m/s^2^)	Acceleration: ±16 g, Gyroscope: ±2000°/s;	Rotation Vector 250 Hz, Linear Acceleration 250 Hz
Stretch Sensor (DMS)	Resistance change measured via voltage divider (330 Ω)	voltage to 1000 Values	250 Hz

**Table 2:** Results of the structured questionnaire evaluating user perception of the smart undershirt (10 = best rating, 0 = worst rating). For the questions marked with an asterisk (*), the scale is inverse, meaning that lower values represent a more positive perception.

Question	Mean ± Standard Deviation
Independent decision on data	5.06 ± 3.96
Feeling of being observed / behavioral change *	3.50 ± 2.31
Number of sensors perceived as disturbing *	2.89 ± 2.22
Perception of compression *	2.17 ± 0.51
Discomfort or irritation *	3.44 ± 2.20
Material pleasantness	7.94 ± 1.51
Ease of putting on the undershirt	6.33 ± 2.00
Ability to perform usual movements	7.78 ± 2.05
Constant awareness of the undershirt *	3.33 ± 2.54
Feeling cold or sweating	6.22 ± 2.58
Breathability of the undershirt	4.82 ± 2.67

## Data Availability

The raw data supporting the conclusions of this article will be made available by the authors on request.
